# Risk factors for recurrence in elderly patients with stage II colorectal cancer: a multicenter retrospective study

**DOI:** 10.1186/s12885-022-09501-8

**Published:** 2022-04-11

**Authors:** Takuki Yagyu, Manabu Yamamoto, Akimitsu Tanio, Kazushi Hara, Ken Sugezawa, Chihiro Uejima, Kyoichi Kihara, Shigeru Tatebe, Yasuro Kurisu, Shunsuke Shibata, Toshio Yamamoto, Hiroshi Nishie, Setsujo Shiota, Hiroaki Saito, Takuji Naka, Kenji Sugamura, Kuniyuki Katano, Yoshiyuki Fujiwara

**Affiliations:** 1 Division of Gastrointestinal and Pediatric Surgery, Department of Surgery, School of Medicine, Faculty of Medicine, Tottori University, 36-1 Nishi-cho, Yonago, 683-8504 Japan; 2 Department of Surgery, Japanese Red Cross Tottori Hospital, Tottori, Japan; 3 Department of Surgery, Tottori Prefectural Central Hospital, Tottori, Japan; 4 Department of Surgery, National Hospital Organization Hamada Medical Center, Hamada, Japan; 5 Department of Surgery, San-in Rosai Hospital, Yonago, Japan; 6 Department of Surgery, Nojima Hospital, Kurayoshi, Japan; 7 Department of Gastroenterological Surgery, Tottori Prefectural Kosei Hospital, Kurayoshi, Japan; 8 Department of Surgery, Masuda Red Cross Hospital, Masuda, Japan; 9 Department of Surgery, National Hospital Organization, Yonago Medical Center, Yonago, Japan; 10 Department of Surgery, Yasugi City Hospital, Yasugi, Japan; 11 Department of Surgery, The Nanbu Town National Health Insurance Saihaku Hospital, Nanbu, Japan

**Keywords:** GNRI, Stage II CRC, Elderly patients

## Abstract

**Background:**

Adjuvant chemotherapy for stage II colorectal cancer (CRC) is considered appropriate for patients with risk factors for recurrence, rather than for all patients uniformly. However, the risk factors for recurrence remain controversial, and there is limited information, especially for elderly patients. The Geriatric Nutritional Risk Index (GNRI) is widely used as a simple nutritional screening tool in the elderly and is associated with cancer prognosis and recurrence. This study aimed to investigate the risk factors for recurrence in the elderly with stage II CRC, focusing on the GNRI.

**Methods:**

We enrolled 348 elderly patients (≥ 75 years) with stage II CRC who underwent curative resection at the Department of Surgery, Tottori University and our 10 affiliated institutions. The patients were divided into GNRI^high^ (≥ 93.465) and GNRI^low^ (< 93.465) groups.

**Results:**

The GNRI^low^ group showed a significantly worse overall survival (OS), cancer-specific survival (CSS), and relapse-free survival (RFS) (*P* < 0.001, *P* < 0.001, and *P* < 0.001, respectively). In a multivariate analysis, GNRI^low^ (hazard ratio [HR]: 2.244, *P* < 0.001), pathologic T4 stage (HR: 1.658, *P* = 0.014), and moderate to severe lymphatic or venous invasion (HR: 1.460, *P* = 0.033) were independent factors affecting RFS. By using these three factors to score the risk of recurrence from 0 to 3 points, the prognosis was significantly stratified in terms of OS, CSS, and RFS (*P* < 0.001, *P* < 0.001, and *P* < 0.001, respectively). The recurrence rate for each score was as follows: 0 points, 9.8%; 1 point, 22.0%; 2 points, 37.3%; and 3 points, 61.9%.

**Conclusions:**

GNRI^low^, pathologic T4 stage, and moderate to severe lymphatic or venous invasion are high-risk factors for recurrence in the elderly with stage II CRC. The scoring system using these three factors appropriately predicted their recurrence and outcome.

## Background

The number of elderly patients diagnosed with colorectal cancer (CRC) continues to increase with the aging of the population worldwide. In fact, approximately 40% of CRC patients are over 75 years [1]. However, because elderly patients are generally excluded from clinical trials, the recommended treatment for this population is more unclear than for non-elderly patients. In particular, elderly patients typically show poor tolerance to chemotherapy, and its administration might worsen their performance status (PS) [2]. In a previous retrospective study, the rate of adjuvant chemotherapy (AC) for patients with stage III CRC decreased dramatically with increasing age: 78% of patients aged 65–69 years, 74% of those aged 70–74, 58% of those aged 75–79 years, 34% of those aged 80–84 years, and 11% of those aged 85–89 years [3]. However, there is a considerable number of the elderly in a good general condition who may benefit from chemotherapy. An analysis of 5489 patients ≥ 75 years of age with resected stage III colon cancer reported a survival benefit of 5-fluorouracil-based AC [4].

The clinical efficacy of AC after curative resection in patients with stage II CRC remains controversial. Currently, guidelines recommend that AC for stage II CRC should be targeted to patients at high risk of recurrence rather than uniformly given to all patients [5–8]. The following variables have been proposed as high-risk factors for recurrence: pathologic T4 stage, perforation, poorly differentiated or undifferentiated adenocarcinoma, venous invasion, lymphatic invasion, and < 12 dissected lymph nodes [9, 10]. However, few studies have focused on risk factors for the recurrence of stage II CRC in elderly patients. Furthermore, most of these proposed risk factors represent only the progression of the tumor itself. Regarding AC for the elderly, it is advisable to identify high-risk factors specific to elderly patients.

In recent years, it has been reported that not only tumor-specific factors but also patient factors related to nutritional status influence survival outcomes in various cancers [11, 12]. The Geriatric Nutritional Risk Index (GNRI) was first reported as an elderly-specific nutritional assessment index to predict nutrition-related risks of morbidity and mortality for hospitalized elderly patients [13]. Recent reports have shown that the GNRI is closely associated with the prognosis of various malignant tumors, including CRC [14], gastric cancer [15], and pancreatic cancer [16]. Interestingly, a low GNRI is not only reported to indicate poor overall survival (OS) due to poor nutritional status but also poor cancer-specific survival (CSS) and relapse-free survival (RFS), which might reflect the state of cancer [16–18].

Therefore, in the present study, we investigated the high-risk factors for recurrence in elderly patients with stage II CRC, focusing on the GNRI.

## Methods

### Patients

The present study included 348 elderly patients aged ≥ 75 years among a total of 713 patients with pathological stage II CRC who underwent radical surgery at the Department of Surgery, Tottori University and our 10 affiliated institutions from January 2007 to December 2017. The eighth edition of the Union for International Cancer Control Tumor, Node, Metastasis staging system was used to determine the clinicopathological characteristics [19]. The ninth edition of the Japanese Classification of Colorectal, Appendiceal, and Anal Carcinoma by the Japan Society for Cancer of the Colon and Rectum was used to evaluate lymphatic invasion and venous invasion [20]. Preoperative data including serum albumin level, C-reactive protein, carcinoembryonic antigen, carbohydrate antigen 19–9, and body weight were measured within 1 month before surgery. Forty-one patients (11.8%) were treated with postoperative adjuvant chemotherapy (uracil/tegafur plus leucovorin, *n* = 15; capecitabine, *n* = 12; uracil/tegafur, *n* = 7; tegafur/gimeracil/oteracil potassium, *n* = 4; fluorouracil plus l-leucovorin, *n* = 1; capecitabine plus oxaliplatin, *n* = 1; fluorouracil/leucovorin plus oxaliplatin, *n* = 1). This study was approved by the Certified Review Board of Tottori University Hospital (18A052) and each institution, and the requirement for informed consent was waived.

### Calculation of the GNRI

The GNRI is a simple index calculated using serum albumin levels (ALB), ideal body weight (IBW), and actual body weight (ABW), which are easily available. The formula for calculating the GNRI is as follows: GNRI = 1.487 × ALB (g/L) + 41.7 × ABW/IBW (kg) [13]. IBW was calculated as 22 × height^2^ (m).

### Statistical analyses

The chi-squared test and Mann–Whitney U test were used to compare the clinicopathological characteristics. The area under the curve (AUC) was calculated by receiver operating characteristic (ROC) analysis. ROC analysis was also used to determine the Youden index for the GNRI. The Kaplan–Meier method was used to generate survival curves, and their differences were examined using the log-rank test. Multivariate analyses were performed using Cox’s proportional hazards model. *P* < 0.05 was considered significant. SPSS software (SPSS for Mac Version 25; IBM Corp., Armonk, NY, USA) was used for statistical analyses.

## Results

**Fig. 1:**
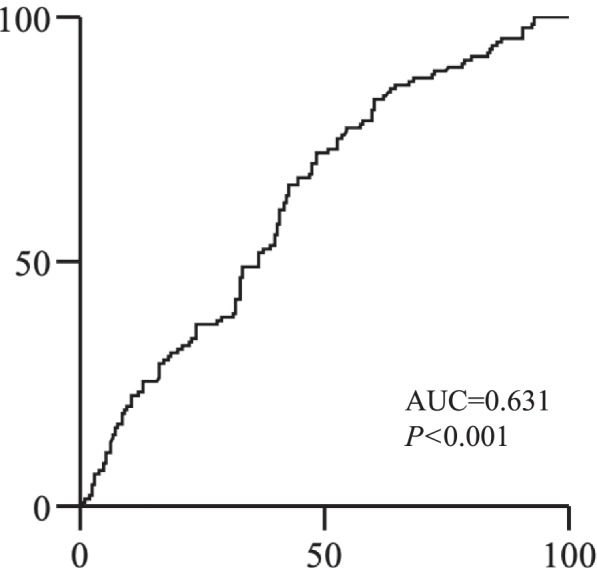
Receiver operating characteristic curves of GNRI for relapse-free survival. GNRI, geriatric nutritional risk index; AUC, area under the curve

We first verified the usefulness of the GNRI in predicting recurrence in the elderly. ROC analysis for RFS showed that the GNRI was considered a useful factor in predicting recurrence (AUC = 0.631; *P* < 0.001; Fig. 1). We then divided patients into GNRI^high^ (≥ 93.465; *n* = 147) and GNRI^low^ (< 93.465; *n* = 201) groups according to the optimal cutoff values determined by ROC analysis. The relationship between GNRI status and clinicopathological factors is shown in Table 1. In addition to body mass index (BMI) and ALB, which are required to calculate the GNRI, there were significant differences between the two groups in age, Eastern Cooperative Oncology Group (ECOG) PS, C-reactive protein, preoperative carcinoembryonic antigen, obstruction, pathologic T stage, and lymphatic invasion.

**Table 1:** Relationship between GNRI status and clinicopathological factors in elderly patients with stage II colorectal cancer

	GNRI^high^ (*n* = 147)	GNRI^low^ (*n* = 201)	*P*value
Age (median)	81 (75–95)	83 (75–98)	< 0.001
Sex			
Male	69 (46.9%)	92 (45.8%)	0.913
Female	78 (53.1%)	109 (54.2%)	
ECOG PS			
0, 1	121 (82.3%)	113 (56.2%)	< 0.001
2, 3, 4	26 (17.7%)	88 (43.8%)	
BMI	23.1 (17.8–28.7)	19.5 (11.7–28.1)	< 0.001
ALB	3.9 (2.8–4.9)	3.1 (1.5–4.8)	< 0.001
CRP	0.16 (0.02–10.30)	0.69 (0.02–34.70)	< 0.001
Preoperative CEA	3.6 (0.8–366.0)	4.9 (1.0–886.3)	0.015
Preoperative CA19-9	11.0 (0–8882.2)	9.1 (0–5782.0)	0.457
Location			
Colon	116 (78.9%)	160 (79.6%)	0.894
Rectum	31 (21.1%)	41 (20.4%)	
Obstruction			
Absent	127 (86.4%)	135 (67.2%)	< 0.001
Present	20 (13.6%)	66 (32.8%)	
Perforation			
Absent	143 (97.3%)	192 (95.5%)	0.569
Present	4 (2.7%)	9 (4.5%)	
Histology ^a^			
tub	135 (91.8%)	176 (87.6%)	0.222
por, muc	12 (8.2%)	25 (12.4%)	
Pathologic T stage ^b^			
T1, T2, T3	133 (90.5%)	152 (75.6%)	< 0.001
T4	14 (9.5%)	49 (24.4%)	
Lymphatic invasion ^c^			
Ly0, 1a	129 (87.8%)	154 (76.6%)	0.008
Ly1b, c	18 (12.2%)	47 (23.4%)	
Vascular invasion ^d^			
V0, 1a	110 (74.8%)	154 (76.6%)	0.706
V1b, c	37 (25.2%)	47 (23.4%)	
Adjuvant chemotherapy			
Absent	128 (87.1%)	179 (89.1%)	0.615
Present	19 (12.9%)	22 (10.9%)	

*GNRI* Geriatric Nutritional Risk Index, *ECOG PS* Eastern Cooperative Oncology Group Performance Status, *BMI* Body mass index, *ALB* Serum albumin level, *CRP* C-reactive protein, *CEA* Carcinoembryonic antigen, *CA19-9* Carbohydrate antigen 19–9

^a^Histology: tub, tubular adenocarcinoma; por, poorly differentiated adenocarcinoma; muc, mucinous adenocarcinoma

^b^Pathologic T stage: T1, Tumor is confined to the submucosa and does not invade the muscularis propria (MP); T2, Tumor invasion to, but not beyond, the MP; T3, Tumor invades beyond the MP. In sites with serosa, the tumor grows into the subserosa. In sites with no serosa, the tumor grows into the adventitia; T4, Tumor invades or perforates the serosa or directly invades other organs or structures

^c^Lymphatic invasion: L1a, Minimal lymphatic invasion; L1b, Moderate lymphatic invasion; L1c, Severe lymphatic invasion

^d^Vascular invasion: V1a, Minimal venous invasion; V1b, Moderate venous invasion; V1c, Severe venous invasion

**Fig. 2:**
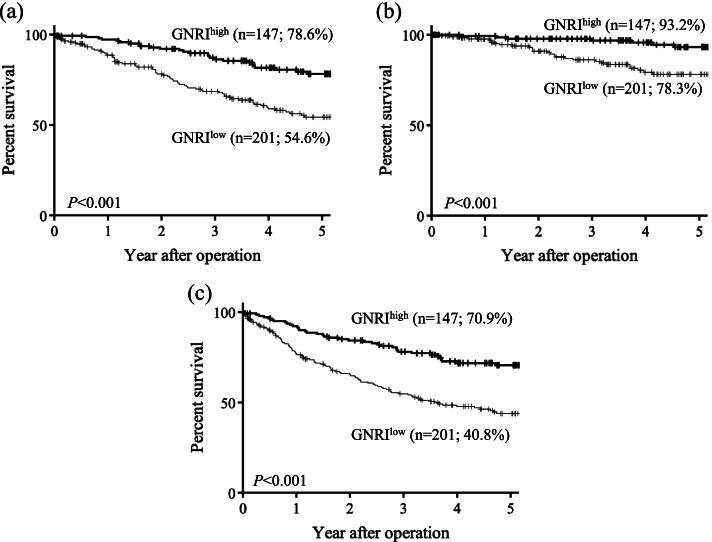
Kaplan–Meier curves according to the GNRI for overall (**a**), cancer-specific (**b**), and relapse-free (**c**) survival. GNRI, geriatric nutritional risk index

The prognosis of the GNRI^low^ group was worse than that of the GNRI^high^ group in terms of 5-year OS (54.6% vs. 78.6%, respectively; *P* < 0.001; Fig. 2a), 5-year CSS (78.3% vs. 93.2%, respectively; *P* < 0.001; Fig. 2b), and 5-year RFS (40.8% vs. 70.9%, respectively; *P* < 0.001; Fig. 2c).

In a multivariate analysis, GNRI^low^ (hazard ratio [HR]: 2.244, 95% confidence interval [CI]: 1.533–3.286, *P* < 0.001), pathologic T4 stage (HR: 1.658, 95% CI: 1.107–2.482, *P* = 0.014), and moderate to severe lymphatic or venous invasion (HR: 1.460, 95% CI: 1.031–2.068, *P* = 0.033) were independent and significant factors affecting RFS (Table 2).

**Table 2:** Univariate and multivariate analyses for relapse-free survival in elderly patients with stage II colorectal cancer

		Univariate analysis			Multivariate analysis		
Variables		HR	95%CI	*P* value	HR	95%CI	*P* value
Obstruction	Present vs. absent	1.202	0.819–1.766	0.347			
Perforation	Present vs. absent	1.863	0.820–4.230	0.137			
Pathologic T stage	T4 vs. others	2.002	1.346–2.979	0.001	1.658	1.107–2.482	0.014
Lymphatic/venous invasion	Ly or V1b/c vs. others	1.586	1.124–2.238	0.009	1.460	1.031–2.068	0.033
Histology	muc or por vs. others	1.101	0.644–1.882	0.726			
CEA	≥ 5.0 ng/ml vs. < 5.0 ng/ml	1.555	1.111–2.174	0.010	1.336	0.950–1.878	0.096
GNRI	< 93.465 vs. ≥ 93.465	2.484	1.708–3.613	< 0.001	2.244	1.533–3.286	< 0.001

*CI* Confidence interval, *HR* Hazard ratio, *CEA* Carcinoembryonic antigen, *GNRI* Geriatric Nutritional Risk Index

See Table 1 for the details of histology, pathologic T stage, and lymphatic/venous invasion

**Fig. 3:**
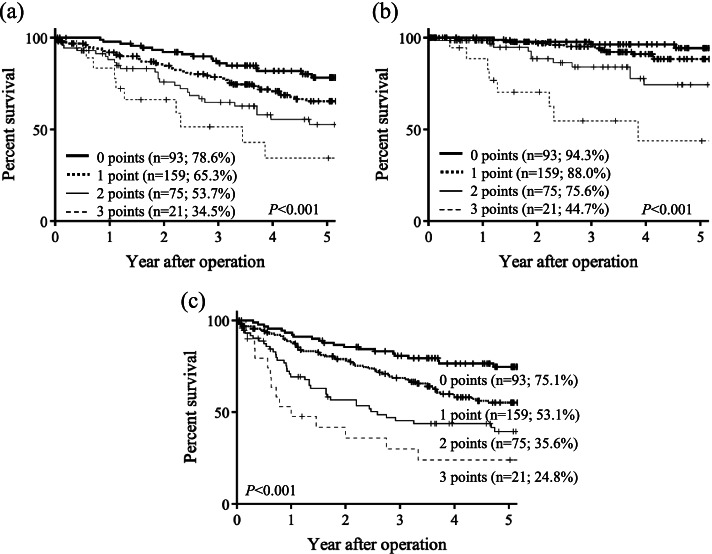
Kaplan–Meier curves according to recurrence prediction scores for overall (**a**), cancer-specific (**b**), and relapse-free (**c**) survival

Previous reports have shown a relationship between the number of risk factors for recurrence and survival in patients with stage II CRC and suggested that AC is more beneficial for patients with multiple risk factors [21–23]. We considered that the adverse effects of AC should be carefully evaluated for elderly patients and that more accurate identification of high-risk patients was warranted. Therefore, we finally developed a scoring system (from 0 to 3 points) to predict recurrence using three independent factors obtained by multivariate analysis. As shown in Fig. 3, the proposed scoring system predicted the patient’s outcome in terms of OS (5-year OS rates, 78.6% vs. 65.3% vs. 53.7% vs. 34.5%, respectively; *P* < 0.001; Fig. 3a), CSS (5-year CSS rates, 94.3% vs. 88.0% vs. 75.6% vs. 44.7%, respectively; *P* < 0.001; Fig. 3b), and RFS (5-year RFS rates, 75.1% vs. 53.1% vs. 35.6% vs. 24.8%, respectively; *P* < 0.001; Fig. 3c). Regarding RFS, the survival curves for each score were generally evenly spaced. Furthermore, the recurrence rate for each score was as follows: 0 points, 9.8%; 1 point, 22.0%; 2 points, 37.3%; and 3 points, 61.9%.

## Discussion

Our study showed that low GNRI level is a prognostic and high-risk factor for recurrence in elderly patients with stage II CRC and that the scoring system using the GNRI, pathologic T4 stage, and lymphatic/venous invasion could stratify patient outcomes in terms of OS, CSS, and RFS.

In recent years, not only the severity of tumor progression but also the patient’s poor nutritional condition have been considered to affect prognosis and recurrence. Several nutritional assessment tools, including the prognostic nutritional index [24], controlling nutritional status [25], and Glasgow prognostic score [26], have been reported as prognostic factors for patients with various cancers. Although these tools are inexpensive and objective, their clinical application is limited because of a lack of consensus in the elderly. In contrast, the GNRI was originally designed to assess nutritional risk for hospitalized elderly patients [13]. Furthermore, this index is calculated using ALB, height, and body weight, which are usually measured before surgery.

Hypoalbuminemia is a known indicator of malnutrition and is closely associated with systemic inflammation and poor immune responses. Tumor-induced systemic inflammation promotes tumorigenesis, invasion, and metastasis via inflammatory mediators, such as tumor necrosis factor-alpha, interleukin-6, and interleukin-10 [27, 28]. Additionally, nutritional deficiencies impair cell-mediated immunity and the function of cytokines and phagocytes, leading to an inadequate anti-tumor immune reaction [29]. Indeed, hypoalbuminemia has been reported as a prognostic factor for immune-checkpoint therapy (ICT) in lung cancer [30], and the Gustave Roussy Immune Score, which is used as a prognostic indicator for ICT, includes low albumin as one of its components [31]. In addition, lower ABW/IBW, which indicates lower BMI, reflects frailty and cachexia and is associated with poor prognosis in elderly patients with cancer [32]. Furthermore, BMI may also be related to tumor immunity. It has been reported that adipose tissue activates cytotoxic T-cells and reduces regulatory T-cells; therefore, a higher BMI leads to a greater effect of ICT [33, 34]. These findings support our results that a low GNRI reflects recurrence and poor prognosis in CRC.

Although nutritional status along with tumor-specific factors is considered important to evaluate patient outcomes, the risk factors for recurrence of stage II CRC reported to date are only related to tumor progression, and to the best of our knowledge, no reports have described nutritional assessment factors. In this study, pathologic T4 stage and lymphatic/venous invasion, which indicate advanced tumor progression, and a low GNRI, which indicates malnutrition, were identified as independent predictors of recurrence, suggesting that both tumor and patient’s nutritional factors have a significant impact on the outcome of elderly patients with stage II CRC.

There are several limitations to this study. First, it is a retrospective cohort study, and the number of cases is not large. Second, surgical techniques, such as the omission of extensive lymph node dissection in the elderly, are not standardized among institutions, which may introduce bias and affect the generalizability of the findings. Third, low GNRI values reflect malnutrition and poor general health; therefore, these patients may not necessarily tolerate chemotherapy. In fact, in our study, a low GNRI was strongly correlated with poor ECOG PS. Fourth, we performed this study with the definition of elderly patients as 75 years and older. Life expectancy has been increasing, and similar studies targeting patients over 80 years of age may be needed.

## Conclusions

GNRI^low^ (< 93.465), pathologic T4 stage, and moderate to severe lymphatic or venous invasion are high-risk factors for recurrence in elderly patients with stage II CRC. The scoring system using these three factors appropriately predicted recurrence rates and outcomes, which may contribute to the decision of appropriate cases for AC.

## Data Availability

The datasets used and analyzed during the current study are not publicly available due to their containing information that could compromise the privacy of research participants but are available from the corresponding author on reasonable request.

## Abbreviations

CRC: Colorectal cancer
PS: Performance status
AC: Adjuvant chemotherapy
GNRI: Geriatric Nutritional Risk Index
OS: Overall survival
CSS: Cancer-specific survival
RFS: Relapse-free survival
ALB: Albumin
IBW: Ideal body weight
ABW: Actual body weight
AUC: Area under the curve
ROC: Receiver operating characteristic
BMI: Body mass index
ECOG: Eastern Cooperative Oncology Group
HR: Hazard ratio
CI: Confidence interval
ICT: Immune-checkpoint therapy
